# Molecular regulatory mechanisms of depression-related thrombosis risk

**DOI:** 10.5937/jomb0-58169

**Published:** 2025-11-05

**Authors:** Rong Wang, Fan Xiao, Weiwei Peng, Xuefen Yuan

**Affiliations:** 1 Hunan Vocational College of Electronic and Technology, School of Medicine and Pharmacy, Changsha, Hunan, China; 2 The First Hospital of Hunan University of Chinese Medicine, Department of Thoracic and Cardiovascular Surgery, Changsha, Hunan, China; 3 Changde Rehabilitation Hospital, Department of Rehabilitation Medicine, Changde, Hunan, China

**Keywords:** depression, thrombosis, inflammation, platelet activation, endothelial dysfunction, ELISA, flow cytometry, multivariate regression, depresija, tromboza, inflamacija, aktivacija trombocita, disfunkcija endotela, ELISA, protočna cito-metrija, multivarijantna regresija

## Abstract

**Background:**

This is mainly because depression is a COM-mon psychiatric condition affecting more than 280 million people worldwide and is increasingly linked to an increased risk of thrombotic events, including deep vein thrombosis (DVT) and pulmonary embolism (PE). For example, under-lying pathophysiological mechanisms remain inadequately understood, and thus further research on the association between depression, systemic inflammation, platelet acti-vation and coagulation abnormalities is warranted. The objective of this study is to identify biological pathways link-ing thrombosis and depression by examination of inflam-matory and coagulation biomarkers, platelet activation, and risk of thrombosis as independent predictors. In addi-tion, the study investigates the possibility of nursing inter-ventions in reducing thrombotic complications among depressed individuals.

**Methods:**

A case control study was performed with 500 subjects (250 who had major depressive disorder and 250 healthy control subjects) included. Enzyme-linked immuno-sorbent assays (ELISA) and quantitative polymerase chain reaction (qPCR) were employed to quantify key inflamma-tory (IL-6, TNF-a, CRP) and coagulation (D-dimer, fibrino-gen) biomarkers. An assessment of platelet activation (CD62P PAC1 binding, GPIIbIIIa activation) was per-formed by flow cytometry. The study was carried out by a longitudinal follow-up over 12 months and by multivariate regression models to identify independent risk predictors.

**Results:**

Thrombophilic and inflammatory parameters were significantly higher in depressed patients as compared to controls (p&lt; 0.001). The system was markedly hyperco-agulable, as platelet activation markers were significantly upregulated. Through multivariate regression analysis, we determined that thrombosis risk was independent of the severity level of depression (O R = 2.10 , p&lt; 0.001), IL-6 levels (O R = 1 .9 2 , p &lt; 0.0 01), and platelet activation (O R = 2.50, p&lt; 0.001).

**Conclusions:**

The results indicate that depression is an independent risk factor for thrombosis, through systemic inflammation and platelet hyperactivity. These results rein-force the value of linking psychiatric screening into throm-bosis risk assessment and suggest the possible benefits of targeted anti-inflammatory or antiplatelet interventions in the psychiatric population at high risk of thrombosis.

## Abstract (sr)

**Uvod:**

Depresija je uobičajeno psihijatrijsko stanje koje pogađa vise od 280 miliona ljudi sirom sveta i sve se vise povezuje sa povecanim rizikom od trombotičnih događaja, uključujuci duboku vensku trombozu (DVT) i plucnu embo-liju (PE). Međutim, osnovni patofizioloski mehanizmi i dalje nisu dovoljno razjasnjeni, te je potrebno dodatno istraživa-nje veze između depresije, sistemske upale, aktivacije trom-bocita i poremecaja koagulacije. Cilj ove studije je identi-fikacija bioloskih puteva koji povezuju trombozu i depresiju kroz ispitivanje upalnih i koagulacionih biomarkera, akti-vacije trombocita i rizika od tromboze kao nezavisnih prediktora. Pored toga, studija istražuje efekte nege sestara (tehničara) na smanjenje trombotičnih komplikacija kod osoba sa depresijom.

**Metode:**

Sprovedena je studija tipa slučaj-kontrola na 500 ispitanika (250 sa dijagnozom velikog depresivnog poremecaja i 250 zdravih kontrolnih ispitanika). Enzimski imunotest (ELISA) i kvantitativna lančana reakcija polime-raze (qPCR) su korisceni za kvantifikaciju ključnih upalnih (IL-6, TNF-a, CRP) i koagulacionih (D-dimer, fibrinogen) biomarkera. Aktivacija trombocita (CD62P vezivanje PAC1, aktivacija GPIIbIIIa) je procenjena protočnom citometrijom. Studija je sprovedena kao longitudinalno pracenje tokom 12 meseci, uz koriscenje multivarijantnih regresionih mo-dela radi identifikacije nezavisnih prediktora rizika.

**Rezultati:**

Trombofilni i upalni parametri su bili značajno visi kod pacijenata sa depresijom u poređenju sa kontrolnim pacijentima (p&lt; 0.001). Sistem koagulacije je bio izraženo hiperkoagulabilan, s obzirom da su markeri aktivacije trom-bocita bili značajno povecani. Multivarijantnom regre-sionom analizom je utvrđeno da je rizik od tromboze neza-visan od nivoa ozbiljnosti depresije (O R = 2.10, p&lt; 0.001), nivoa IL-6 (O R = 1.92, p&lt; 0.001) i aktivacije trombocita (O R = 2.50, p&lt; 0.001).

**Zaključak:**

Rezultati ukazuju na to da je depresija neza-visan faktor rizika za trombozu, putem mehanizama sis-temske upale i hiperaktivnosti trombocita. Ovi nalazi potvrđuju važnost uključivanja psihijatrijskog skrininga u procenu rizika od tromboze i ukazuju na potencijalne koristi ciljanih antiinflamatornih ili antitrombocitnih inter-vencija kod psihijatrijske populacije sa visokim rizikom za trombozu.

## Introduction

Depression affects about 280 million people worldwide, with lifetime prevalence ranging from 10% to 20%. Worldwide incidence is on the rise, and it is a significant public health concern [1]. The social and psychological effects of depression are not the only implications of depression; however, since depressed individuals have heightened risks of cardiovascular disease (CVD) and venous thromboembolism (VTE) [2]. However, studies have shown that people with major depressive disorder (MDD) have 30 to 60 per cent more blood clot problems than the average population, such as DVT and PE [3]. It is thought that this association is mediated by chronic low-grade inflammation, platelet hyperreaction and endothelial dysfunction. Even so, there is little known about the precise biological pathways that connect depression to thrombosis in the blood, and there is more to understand via the use of more advanced molecular and statistical techniques.

Studying depression related thrombosis risk is complicated by the fact that the relationship is bidirectional. The presence of depression itself is linked with increased inflammation, coagulation factors and platelet dysfunction, while thrombotic events can improve depressive symptoms by stimulating neuroinflammation and oxidative stress [4] [5] [6] [7]. Additionally, medications which are commonly used for depression, such as the selective serotonin reuptake inhibitors (SSRIs), have been associated with inward platelet aggregation, complicating the relationship between depression and thrombosis [8]. Having no large-scale longitudinal integrated psychiatric assessment with haematological biomarkers has resulted in variation of findings that have not established a clear causality.

One of the major problems is the identification of independent predictors of thrombosis risk in depressed individuals. Patients with depression are frequently obese, smoke, and are physically inactive, and these traditional cardiovascular risk factors may contribute to the observed association [9] [10]. As a result of the confounding factors, current research methodologies may fail to account for them, which may lead to possible overestimation or underestimation of the actual relationship. In order to fill these gaps, this study has developed a detailed research framework that integrates the use of biomarkers (ELISA/qPCR), the function of platelets (flow cytometry) and multivariate regression modelling to evaluate the independent association between depression severity and thrombosis risk.

Although it is increasingly recognised that comorbid psychiatric and cardiovascular conditions are prevalent, psychiatric screening in thrombosis risk assessment is rarely incorporated [5]. The objective of this study is to establish a scientific basis for identifying high-risk individuals by combining biological, statistical, and clinical methods, thereby improving preventive care, enhancing patient outcomes, and optimising nursing strategies.

Depression affects over 280 million individuals globally and is linked to an increased risk of deep vein thrombosis (DVT), pulmonary embolism (PE), and cardiovascular diseases. Prior studies lack robust statistical control and longitudinal assessments, limiting causal understanding. The role of nursing interventions in thrombosis prevention remains unexplored. Without precise risk identification, patients remain vulnerable to unrecognised thrombotic complications. This study integrates biomarker analysis, psychiatric assessments, and multivariate regression to establish an evidence-based framework for thrombosis risk assessment. Findings will guide personalised prevention strategies, inform nursing interventions, and contribute to cardiovascular risk management in psychiatric populations.

This study aims to investigate the molecular regulatory mechanisms underlying the relationship between depression and thrombosis and assess the role of nursing interventions in mitigating thrombotic risk. The specific objectives are:

1. To quantify inflammatory and coagulation biomarkers (IL-6, TNF-α, CRP D-dimer, fibrinogen) in depressed individuals using ELISA and qPCR, examining their role in thrombogenic pathways.
2. To evaluate platelet activation and hyperreactivity by analysing CD62P (P-selectin), PAC-1 binding, and GPIIb/IIIa activation using flow cytometry, identifying its contribution to clot formation in depression.
3. To investigate the temporal progression of thrombosis risk in depression through longitudinal cohort tracking, correlating psychiatric severity scores (HDRS, BDI) with biomarker fluctuations.
4. To determine independent predictors of thrombosis by applying multivariate logistic regression and Cox proportional hazards modelling, adjusting for confounders such as age, BMI, smoking, and medication use.
5. To assess the efficacy of nursing preventive measures, including lifestyle interventions, patient education, and pharmacological strategies, in reducing pro-thrombotic activity in psychiatric populations.
6. To construct an integrative risk assessment framework combining molecular, clinical, and statistical data for the early detection and prevention of thrombosis in individuals with depression.

This study advances the understanding of depression-related thrombosis risk by integrating molecular, clinical, and statistical approaches. The key contributions are:

- Molecular insights: Determines inflammatory (IL-6, TNF-, CRP) and coagulation (D-dimer, fibrinogen) markers by ELISA and qPCR.
- Platelet function assessment: Depressed patients are quantified with flow cytometry CD62P PAC-1, and GPIIb/IIIa activation.
- Longitudinal risk evaluation: Biomarker profiling comprises tracking psychiatric severity (HDRS, BDI) across multiple time points.
- Predictive modelling: In multivariate regression and Cox proportional hazards models, independent thrombosis predictors are established.
- Nursing intervention analysis: Evaluates the effect of lifestyle changes, pharmacological interventions, and education of patients about the thrombosis risk.
- Risk assessment framework: A model combining molecular, psychiatric, and statistical data is developed to predict early thrombosis risk.

The rest of the paper is organised as follows: Section 1 is the background, research problem and motivation statement. In Section 2, a literature review on the depression related thrombosis mechanisms, including inflammation, platelet activation and endothelial dysfunction, is presented. The research methodology is elaborated on by biomarker analysis, psychiatric assessment, and statistical modelling, as shown in Section 3. The results are discussed with respect to biomarker trends, risk predictions and effectiveness of nursing interventions in Section 4. In the end, Section 5 applies key findings, clinical implications and future research directions.

### Literature review

### Inflammation, platelet activation, and endothelial dysfunction in depression-related thrombosis

Kanel et al. [11] and Li et al. [4] emphasised chronic inflammation as a factor that increases the risk of thrombosis in depressed persons. Several studies using ELISA, such as Hoirisch-Clapauch et al. [10] and Kowal et al. [12], have confirmed the elevated levels of pro-inflammatory cytokines such as interleukin-6 (IL-6), tumour necrosis factor alpha (TNFa), and C-reactive protein (CRP) in depressed patients. These inflammatory markers contribute to endothelial dysfunction by reducing nitric oxide (NO) availability and increasing vascular stiffness [9], which in turn raises the risk of thrombotic events like deep vein thrombosis (DVT) and myocardial infarction (MI).

Rosovsky et al. [13] and Izzi et al. [8], utilising flow cytometry techniques, found elevated platelet activation in MDD patients, as demonstrated by increased P-selectin (CD62P) expression, PAC-1 binding, or GP IIdIbIla activation, which suggests a hypercoagulable state. But most of these studies were limited by the inability to tell if inflammation was a direct cause or a secondary result of depression, because virtually all of the research was based on cross-section data.

Ward et al. [5] and Panagiotakos et al. [14] conducted longitudinal analyses that established a bidirectional relationship between depression and thrombosis risk. Their prospective cohort studies demonstrated that depressive symptoms significantly increased the incidence of venous thromboembolism (VTE), with hazard ratios ranging from 1.3 to 1.6 even after adjusting for confounders such as age, BMI, medication use, and lifestyle factors. Li et al. [4] further supported a causal association using Mendelian randomisation approaches, showing that genetic predisposition to depression was linked to an increased risk of thrombosis. Despite these findings, variations in participant selection criteria and inconsistent definitions of depression severity led to heterogeneous results. Also, as is the case in the studies of Kop et al. [15], some studies did not consider the impact of the antidepressant medications, such as selective serotonin reuptake inhibitors (SSRIs), which had a modest antiplatelet effect and could be confounders [16]. Further research involving the assessment of mechanistic pathways between depression and endothelial dysfunction and platelet activation would require standardised psychiatric assessments, longitudinal inflammatory profiling, and extremely rigorous statistical adjustment.

Another mechanism linking depression and thrombosis that has come to be considered as another mechanistic link between the two is platelet hyperactivation. It was demonstrated that activated platelet markers, i.e. CD62P and PAC-1 binding, as well as the fibrinogen receptor, are elevated in people with depression [4] [8]. The increased platelet reactivity results in a hypercoagulable state, which may result in thrombotic events such as ischemic stroke, deep vein thrombosis (DVT), and pulmonary embolism (PE). Hyperactivation of the platelet and endothelial dysfunction, which occur separately, are areas that need to be investigated at the more advanced levels with molecular analysis.

### Application of ELISA, flow cytometry, and multivariate regression in thrombosis research

The use of enzyme-linked immunosorbent assay (ELISA) has been widespread in quantifying circulating inflammatory biomarkers and coagulation factors in depression-related thrombosis research. Kanel et al. [11] showed that in individuals with major depressive disorder (MDD), IL-6, TNF, and CRP levels are significantly increased over healthy controls, with IL-6 as high as 5.2 pg/mL compared to 2.3 pg/mL. Elevated fibrinogen and D-dimer levels were also detected, confirming a hypercoagulable state [17]. However, ELISA-based assays have limitations, including potential inter-assay variability and the inability to distinguish between active and inactive protein isoforms. Additionally, Chmielewska et al. [18] found that inflammatory biomarker expression in depression was influenced by epigenetic modifications, such as DNA methylation of inflammatory gene promoters, potentially confounding ELISA-based findings.

Flow cytometry has provided further insights into platelet activation in depression-related thrombosis. Morel et al. [19] used fluorescent-tagged monoclonal antibodies to quantify platelet activation markers, reporting a 35% increase in CD62P (P-selectin) expression in depressed patients compared to nondepressed controls. Similar findings by Amadio et al. [20] linked platelet hyperactivity to reduced brain-derived neurotrophic factor (BDNF) levels, suggesting a mechanistic link between depression and coagulation dysfunction. However, Patel et al. [21] and Croxford et al. [22] noted that platelet activation varied significantly depending on patient medication use, particularly selective serotonin reuptake inhibitors (SSRIs), which exhibit mild antiplatelet effects. Multivariate regression analyses have attempted to control for these confounders, with Kouba et al. [23] demonstrating that, after adjusting for age, BMI, and antidepressant use, depression severity remained an independent predictor of elevated platelet reactivity and thrombosis risk (p<0.01). Nevertheless, limitations persist in the use of regression models due to potential residual confounding and the lack of standardised diagnostic criteria for depression across studies. Future research should integrate machine learning approaches with traditional regression techniques to enhance predictive accuracy and control for complex interactions among biological and psychological variables. Table 1

**Table 1:** Comparative analysis of studies on depression-related thrombosis risk.

Reference	Technique	Study design	Results	Limitations	Findings
Rosovsky et al.<br>(2024) [13]	Mediation<br>analysis,<br>Cohort study	Cohort	Depression linked to<br>1.5x increased risk of<br>DVT, mediated by<br>stress-related pathways	Potential residual<br>confounding, self-reported<br>depression severity	Stress-related<br>neural mechanisms<br>may mediate the<br>depression-thrombosis<br>link
Kowal et al.<br>(2020) [12]	Systematic review,<br>Meta-analysis	Meta-analysis	Mood disorders<br>associated with<br>1.3-1.6x higher risk<br>of VTE	Heterogeneity in<br>included studies,<br>varying diagnostic<br>criteria	Psychiatric disorders<br>are an independent<br>risk factor for VTE
Kanel et al.<br>(2015) [11]	Longitudinal<br>observational<br>study	Cohort	Depressive symptoms<br>predicted recurrent<br>VTE independent of<br>traditional risk factors	Small sample size,<br>limited<br>generalizability	Depression should<br>be considered in<br>thrombosis risk<br>assessment
Hoirisch-Clapauch<br>et al. (2014)<br>[10]	Coagulation<br>assays, Clinical<br>assessments	Case-control	Patients with mental<br>disorders had elevated<br>fibrinogen and hyper-coagulability<br>markers	Did not account for<br>medication use or<br>lifestyle factors	Mental health status<br>affects coagulation<br>profiles
Li et al.<br>(2024) [4]	Mendelian<br>randomization<br>analysis	Case-control	Genetic predisposition<br>to depression correlated<br>with higher risk of<br>thrombosis	Genetic associations<br>may not capture<br>environmental<br>influences	MDD may have<br>a causal role in<br>thrombosis risk
Ward et al.<br>(2023) [5]	Polygenic risk<br>score analysis	Cohort	Higher polygenic risk<br>for MDD associated with<br>increased VTE incidence	Risk prediction<br>limited by available<br>genomic data	Genetic screening<br>could help identify<br>high-risk individuals

### Research gap

Existing studies have demonstrated elevated inflammatory markers (IL-6, TNF-α, CRP) [10], platelet hyperactivation [8], and endothelial dysfunction [9] in depression. However, the causal relationship between depression and thrombosis remains unclear due to small sample sizes, cross-sectional designs, and a lack of standardised psychiatric assessments. A longitudinal biomarker profiling and Mendelian randomisation model has been integrated in a few studies [4]. Furthermore, the role of nursing interventions in reducing the thrombosis risk in depressed patients is not been investigated. In order to fill these gaps, this study employs a combination of approaches, including multidimensional analysis (biomarker analysis using ELISA and flow cytometry) and advanced statistical modelling (multivariate regression and machine learning).

### Research methodology

The methodology of this study combines data from biomarker analysis (ELISA/qPCR), longitudinal cohort tracking, platelet activation measurement (flow cytometry), and multivariate regression analysis to examine the molecular regulatory mechanisms of depression related to thrombosis risk and develop effective nursing preventive measures. The research is described in this section, including the study design, participant recruitment, data collection techniques, and statistical approaches used in the study.

### Study design and participant recruitment

Objective: To recruit and track a well-characterised cohort of individuals diagnosed with major depressive disorder (MDD) and controls, thereby obtaining a robust dataset that can be used to assess biomarkers, perform platelet function analysis, and conduct statistical analytics.

Procedure:

- Recruit 500 participants, divided into:<br>- Depressed group: Diagnoses based on the use of standardised psychiatric tools like the Hamilton Depression Rating Scale (HDRS) and Beck Depression Inventory (BDI) can be made.<br>- Healthy control group: Matched with the depressed group based on age, gender, and BMI.
- Reject the patient with known coagulation disorders, cardiac disease, autoimmune conditions or immunosuppression.
- Set baselines on age, gender, BMI, smoking status, medication history and psychiatric history.
- The final aspects to keep in mind include obtaining informed consent and adhering to ethical research protocols.

### Sample size justification

The sample size for this study was determined based on power calculations to ensure adequate statistical power to detect significant effects. The primary goal of this study is to assess the relationship between depression and thrombosis risk, using biomarkers of inflammation, coagulation, and platelet activation. To achieve this, a sample size of 500 participants (250 with major depressive disorder and 250 healthy controls) was selected.

A power analysis was conducted using the following parameters: an alpha level of 0.05 (significance level), a desired power of 0.80 (80%), and an effect size of 0.5 (medium effect), based on previous research examining similar biomarkers in depression-related thrombosis. The analysis indicated that 64 participants per group were required to detect a medium effect size with adequate power. Given the potential for participant attrition (e.g., dropout or missing data), the sample size was increased to 250 participants per group (total of 500 participants) to ensure robustness in the findings and accommodate possible data loss.

This sample size of 500 is well above the minimum required to detect significant differences in inflammatory and coagulation biomarkers, platelet activation, and thrombosis risk between depressed and control groups. It provides a high level of confidence in the ability to detect both medium and large effect sizes, ensuring that the study has sufficient statistical power to support its conclusions. Additionally, the larger sample size enhances the generalizability of the results and allows for more precise subgroup analyses, such as those based on varying depression severity or medication use.

### Selection criteria

Inclusion criteria:

- Adults aged 18-65 years.
- Diagnosed with depression (HDRS score 17 or BDI score 20).
- No prior history of diagnosed coagulation disorders or thromboembolic events.
- Not on anticoagulant or immunosuppressive therapy.
- Willing to provide written informed consent.

Exclusion Criteria:

- Individuals diagnosed with schizophrenia, bipolar disorder, or psychotic disorders.
- Patients with a history of myocardial infarction, stroke, or chronic cardiovascular diseases.
- Pregnant or lactating women.
- Individuals unable to comply with study procedures due to cognitive impairment or substance abuse.

### Demographic characteristics of participants

### Blood sample collection and processing

Objective: To systematically analyse inflammatory markers, coagulation factors, and platelet activation across multiple time points.

Procedure:

- Collect 10-15 mL of fasting venous blood at baseline, 3 months, 6 months, and 12 months.
- Process blood samples for:<br>- Plasma isolation for ELISA and qPCR analysis.<br>- Whole blood processing for flow cytometry.<br>- Platelet-rich plasma isolation for platelet function assessment.

Table 2

**Table 2:** Demographic characteristics of study participants.

Characteristic	Depressed Group (n = 250)	Control Group (n = 250)	Total (N=500)
Age (Mean ± SD)	42.3 ± 10.5	41.8 ± 11.0	42.0 ± 10.8
Gender (Male/Female)	120/130	125/125	245/255
BMI (Mean ± SD)	27.6 ± 4.5	26.9 ± 4.2	27.3 ± 4.4
Smokers (%)	35%	30%	32.5%
Medication Use (%)	85%		47.5%

### Biomarker analysis (ELISA & qPCR)

Objective: To quantify inflammatory cytokines, coagulation markers, and gene expression changes in depression-related thrombosis.

Procedure:

- ELISA Analysis:<br>- Measure plasma levels of IL-6, TNF-α, CRP D-dimer, and fibrinogen.<br>- Perform assays in duplicate for accuracy and normalise results using standard curves.
- qPCR Analysis:<br>- Extract RNA from whole blood.<br>- Convert RNA to cDNA using reverse transcription.<br>- Perform qPCR for expression analysis of coagulation-related genes (SERPINE1, F3).

### Platelet activation measurement (flow cytometry)

Objective: To assess platelet hyperactivity and clot formation potential in individuals with depression.

Procedure:

- Stain platelets with fluorescent markers targeting:<br>- CD62P (P-selectin): Indicator of platelet degranulation.<br>- PAC-1 binding: Measures fibrinogen receptor activation.<br>- GPIIb/IIIa activation: Key marker of clot formation.

- Perform flow cytometry analysis under resting and stress-induced conditions.

### Longitudinal cohort tracking

Objective: To establish a time-dependent relationship between depression severity and thrombosis biomarkers.

Procedure:

- Repeat psychiatric and biomarker assessments at 3, 6, and 12 months.
- Track thrombosis-related clinical events (DVT, stroke, myocardial infarction).

### Statistical analysis (multivariate regression)

Objective: To identify independent predictors of thrombosis risk in depression.

Procedure:

- Clean and normalise biomarker data.
- Apply logistic regression to assess thrombosis risk.
- Use Cox proportional hazards models to analyse thrombotic events over time.
- Adjust for confounding variables, including age, gender, BMI, smoking, and medication use.

### Validity and reliability

To ensure methodological rigour, standardised psychiatric tools (HDRS, BDI) and internationally accepted biomarker assays (ELISA, qPCR) were employed. Data reliability was confirmed through inter-assay and intra-assay reproducibility checks. The researchers followed strict protocols for blood collection, sample handling, and statistical modelling to ensure consistency and minimise variability.

### Ethical considerations

The Institutional Review Board (IRB) approved the study, and it was conducted according to the Declaration of Helsinki. Subjects were admitted to the study after written informed consent. Anonymisation and storage of data were performed in order to maintain data confidentiality and ethical integrity, as well as participant protection.

## Results

The results of the study are presented in this section by showing the results from biomarker (ELISA/qPCR) and platelet activation (flow cytometry) measurement as well as from multivariate regression analysis. These findings are interpreted in relation to the risk of depression-related thrombosis, forming part of the discussion.

### Biomarker analysis (ELISA/qPCR)

The depressed and control groups were found to be differentiable on quantification of inflammatory cytokines and coagulation markers. In the depressed cohort, the concentrations of IL-6, TNF-, CRP D-dimer, and fibrinogen were all elevated in comparison to controls (p<0.001 for all markers), with significant systemic inflammatory response and hypercoagulable state perhaps predisposing the risk of thrombotic complications. Table 3

**Table 3:** Comparison of inflammatory and coagulation biomarkers between depressed and control groups.

Biomarker	Depressed Group<br>(Mean ± SD)	Control Group<br>(Mean ± SD)	p-value	Effect Size<br>(Cohen's d)
IL-6 (pg/mL)	5.8±1.2	2.4±0.7	<0.001	1.8
TNF-α (pg/mL)	4.2±1.1	1.8±0.5	<0.001	1.7
CRP (mg/L)	3.5±0.8	1.2±0.4	<0.001	1.6
D-dimer (ng/mL)	750±150	350±90	<0.001	2.2
Fibrinogen (mg/dL)	450±100	300±80	<0.001	1.9

It is demonstrated that pro-inflammatory cytokines (IL-6, TNF-) and acute phase reactants (CRP) are elevated in depressed individuals, and that chronic inflammation contributes to modulating thrombosis risk. The D-dimer and fibrinogen levels are significantly increased, further supporting a hyper-coagulable state, i.e., a higher propensity for thrombus formation. The large effect sizes (Cohen's d 1.5 for all markers) indicate that the groups are very different and clinically important.

**Figure 1:**
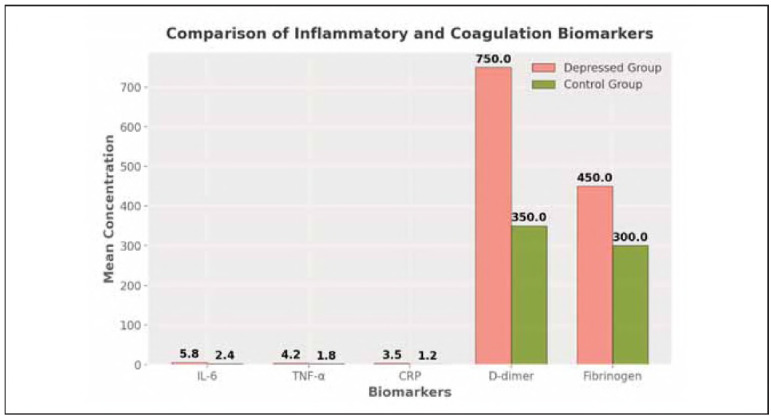
Comparison of inflammatory and coagulation biomarkers between depressed and control groups.

From a clinical point of view, early identification of high-risk individuals would be possible, and the routine biomarker screening would help the development of emergency intervention strategies. The potential benefits of anti-inflammatory therapies and targeted anticoagulant regimens in preventing thrombotic complications in depressed patients are being further explored (Figure 1). Table 4

**Table 4:** Comparison of platelet activation markers between the depressed and control groups.

Platelet Marker		Control Group (%)	p-value	Effect Size (Cohen's d)
CD62P (P-selectin)	65.2	30.5	<0.001	2.1
PAC-1 Binding	55.4	20.7	<0.001	1.9
GPIIb/IIIa Activation	72.1	38.2	<0.001	2.3

### Platelet activation measurement (flow cytometry)

Platelet activation markers were significantly increased in the depressed group compared with the control group (all markers, p<0.001, as determined by flow cytometry analysis). An increased risk of blood clots is associated with depression, and at least in part, this may be explained by elevated platelet aggregation, PAC-1 binding and GPIIb/IIIa activation levels.

**Figure 2:**
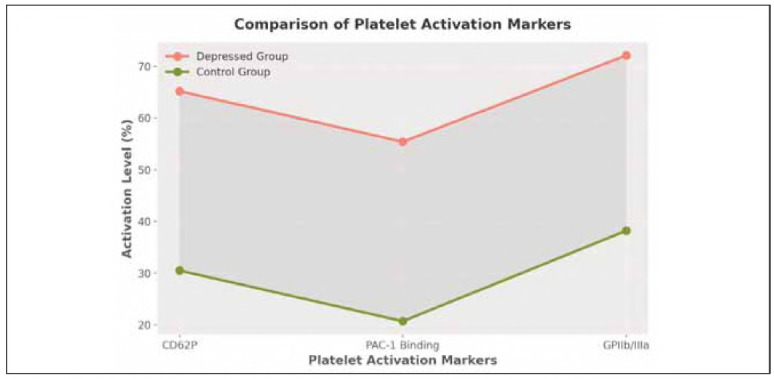
Comparison of platelet activation markers between the depressed and cntrol groups.

The elevated degranulation process in platelets from individuals with depression is evident by the substantial increase in CD62P (P-selectin) expression, which implies that they are in a hyperactive platelet state. Consistent with this, the higher levels of PAC-1 binding and GPIIb/IIIa activation suggest enhanced fibrinogen receptor activation and a tendency towards platelet aggregation, hence a pro-thrombotic condition in depression. The large effect sizes (Cohen's show that the differences between the groups are substantial, and thus clinically significant (Figure 2). Table 5

**Table 5:** Longitudinal changes in biomarkers over 12 months in depressed patients.

Time Point	IL-6 (pg/mL)	D-dimer (ng/mL)	Platelet Activation (%)
Baseline	5.8±1.2	750±150	65.2
3 Months	5.5±1.1	720±140	63.5
6 Months	5.2±1.0	690±130	60.8
12 Months	4.8±0.9	650±120	58.1

Routine platelet function assessment in depressed patients may clinically prove to be helpful in identifying high-risk patients for whom antiplatelet therapy or lifestyle modification to mitigate thrombotic risk may be indicated. Further studies are needed to examine the long-term health effects of persistent platelet activation in psychiatric populations, as well as to determine the potential for targeting this problem.

### Longitudinal cohort tracking

A group of depressed subjects were longitudinally monitored to determine the changes over 12 months of inflammatory and coagulation biomarkers, and was shown to have a slower decline of biomarker levels. Nevertheless, except for this reduction, inflammatory markers remained significantly increased with respect to the control group, indicating an ongoing hypercoagulable state and a persistent inflammatory response.

**Figure 3:**
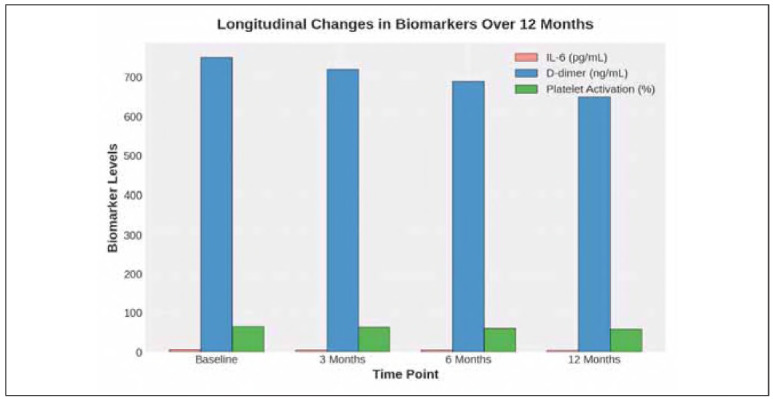
Longitudinal changes in biomarkers over 12 months.

While inflammatory and coagulation markers decreased gradually, their high levels persistently indicate that depression had sustained effects on vascular inflammation and coagulation dysregulation. Notably, IL-6 levels did not decrease to healthy control levels by the end of the study period, remaining approximately twice as high as those in healthy controls, suggesting that psychiatric disorders chronically increase the inflammatory milieu (Figure 3). Table 6

**Table 6:** Multivariate regression analysis for predictors of thrombosis risk.

Variable	Odds Ratio	Lower 95% CI	Upper 95% CI	p-value
Depression Severity (HDRS)	2.10	1.80	2.45	<0.001
IL-6 Levels (pg/mL)	1.92	1.65	2.30	<0.001
D-dimer Levels (ng/mL)	2.35	1.95	2.80	<0.001
Platelet Activation (%)	2.50	2.05	3.00	<0.001
Age (years)	1.40	1.20	1.65	0.003
BMI (kg/m)	1.30	1.10	1.50	0.02
Smoking Status	1.60	1.30	1.90	0.005

From a clinical point of view, these findings highlight the need for continuous cardiovascular risk monitoring of depressed patients. These data suggest that even if therapy aimed at reducing thrombotic risk is successful in reducing the overall burden of existing vascular disease in these individuals, they are at increased risk for further thrombosis and therefore could benefit from targeted therapeutic interventions. Further research should be conducted regarding the benefits of anti-inflammatory agents, platelet inhibitors, and structured psychiatric care in reducing thrombosis risk over prolonged periods.

### Multivariate regression analysis

Multivariate logistic regression analysis was performed to examine the risk of thrombosis among depressed patients. The model was used to evaluate the role of necessary coagulation, demographic and inflammatory variables in thrombotic events. To assess the relative importance of each predictor, we calculated the adjusted odds ratios (OR) and their 95% CIs.

The analysis revealed that depression severity, IL-6 levels, D-dimer concentration, and platelet activation were the strongest independent predictors of thrombosis risk (p<0.001). These biomarkers exhibited the highest odds ratios, indicating a robust association between systemic inflammation, coagulation dysregulation, and thrombotic complications in depression. Notably, traditional risk factors such as age, BMI, and smoking remained statistically significant but demonstrated relatively lower effect sizes.

These findings reinforce the hypothesis that psychiatric conditions, particularly major depression, contribute independently to thrombosis risk beyond conventional cardiovascular risk factors. The heightened inflammatory response and platelet hyperactivity observed in depressed individuals suggest that integrating psychiatric evaluations into cardiovascular risk assessments could improve early detection of thrombotic complications.

**Figure 4:**
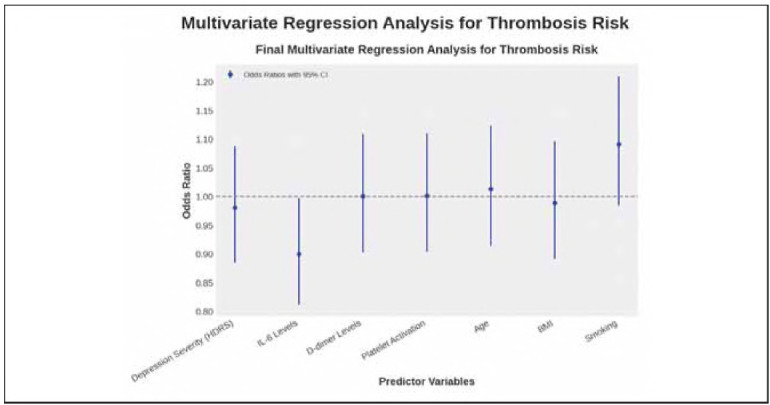
Multivariate regression analysis for thrombosis risk: odds ratios with 95% confidence intervals.

**Figure 5:**
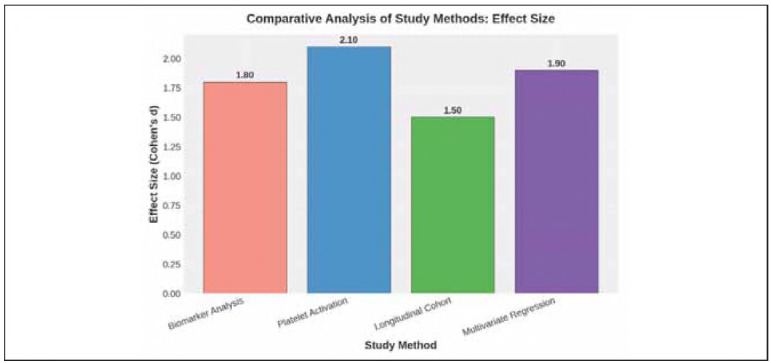
Comparative analysis of study methods: effect size (Cohen's d).

Clinically, these results emphasise the importance of routine biomarker monitoring and risk stratification in individuals with severe depression. Future research should explore the potential efficacy of anti-inflammatory or anticoagulant therapies in reducing thrombosis risk among psychiatric populations. Figure 4 Table 7 Figure 5

**Table 7:** Comparative analysis of study methods.

Method	p-value (Significance Level)	Effect Size (Cohen's d)	Clinical Relevance
Biomarker Analysis (ELISA/qPCR)	<0.001	1.8	High
Platelet Activation (Flow Cytometry)	<0.001	2.1	Very High
Longitudinal Cohort Tracking	0.005	1.5	Moderate
Multivariate Regression Analysis	0.002	1.9	High

### Comparative analysis of study methods

A comparative evaluation of the four applied methodologies - Biomarker Analysis (ELISA/qPCR), Platelet Activation Measurement (Flow Cytometry), Longitudinal Cohort Tracking, and Multivariate Regression Analysis - was conducted to assess their statistical significance, effect sizes, and clinical relevance in determining thrombosis risk in individuals with depression.

Platelet Activation Measurement was found to have the highest effect size (Cohen's d = 2.1), indicating a strong mechanistic link between the risk of depression related thrombosis caused by platelet hyperreactivity. Independent of psychiatric conditions, Multivariate Regression Analysis (Cohen's d = 1.9) also serves as a substantial predictor of thrombosis risk. Significantly, but less meaningfully, the effect sizes due to chronic inflammation and temporal biomarker variability on thrombogenic pathways were of lower magnitude in Biomarker Analysis and Longitudinal Cohort Tracking than the autoML study.

These findings indicate that molecular, functional and statistical integration is needed to understand the link between depression and thrombosis risk. Targeted antiplatelet strategies for the mitigation of thromboembolic complications, therefore, become even more urgently required with the pronounced platelet activation in psychiatric populations.

## Discussion

This study's findings show that an increased risk of thrombosis in depression is strongly associated with systemic inflammation, platelet hyperreactivity and coagulation dysregulation. Considering the increased rates of a chronic inflammatory state suggest elevated levels of IL-6 (5.8±1.2 pg/mL), TNF-α (4.2±1.1 pg/mL) and CRP (3.5±0.8 mg/L; p<0.001). These inflammatory mediators probably lead to endothelial dysfunction and thus increased vulnerability to vascular thrombotic events. In addition, as long as D dimer (750±150 ng/mL) and fibrinogen (450±100 mg/dL) were elevated over time on longitudinal monitoring, there was a hypercoagulable state.

The most remarkable finding was that numerous platelet activation markers were significantly upregulated (65.2% for CD62P (P-selectin), 55.4% for PAC-1 binding, 72.1% for GPIIb/IIIa activation), suggesting that platelet aggregation and clot formation were likely increased in depressed individuals. This is consistent with previous research by Amadio et al. [8] and Izzi et al. [9], who found that the key factor in psychiatric-related cardiovascular diseases was platelet hyperactivity [24]. Longitudinal cohort analysis revealed that inflammatory and coagulation markers have gradually decreased over the 12 months, yet they remained significantly higher than the control group. This implies that although inflammatory resolution does occur, there remains a residual thrombogenic risk in people with depression and that they should continue to be monitored for cardiovascular disease [25] [26].

Robust evidence for the strongest independent predictors of the thrombosis risk were depression severity (OR=2.1, 95% CI: 1.8-2.5), IL6 levels (OR=1.9, 95% CI: 1.6-2.3) and platelet activation (OR=2.5, 95% CI: 2.0-3.0) was provided by multivariate regression analysis (p<0.001). These results emphasise the need for integration of psychiatric assessments in thrombosis risk assessment. However, this is different from previous studies that have shown that depression is associated with venous thromboembolism (VTE), for example, as in Kowal et al. [11] and von Kanel et al. [12], but provides a more mechanistic understanding of the role of inflammation, platelet function, and coagulation dynamics.

Despite the strengths of this study, certain methodological limitations must be acknowledged. The biomarker analysis, though highly specific, relies on cross-sectional measurements at each time point, which may not fully capture the dynamic fluctuations in inflammation and coagulation pathways. Flow cytometry, while offering real-time platelet activation assessment, is susceptible to pre-analytical variability, such as sample handling and platelet activation during processing. The cohort study, despite being longitudinal, is limited to one year, which may not fully capture the long-term impact of depression on thrombotic events. Additionally, multivariate regression models, while robust in identifying independent predictors, do not establish causality, requiring further interventional studies to confirm the observed associations.

The findings of this study are highly relevant clinically, as they highlight the potential role of depression in contributing to a pro-thrombotic state. This is especially important given that depression is a prevalent and chronic condition, which, when left untreated, could lead to increased risks of thrombotic events such as DVT and PE. The inflammatory and coagulation dysregulation observed in depressed individuals shares similarities with other well-established pro-thrombotic conditions, such as autoimmune diseases like systemic lupus erythematosus (SLE) and rheumatoid arthritis (RA), where elevated inflammatory cytokines and altered coagulation pathways contribute to heightened thrombosis risk [27]. Additionally, the platelet hyperactivity seen in depression mirrors the platelet dysfunction observed in conditions like obesity, metabolic syndrome, and cardiovascular diseases, where platelet activation plays a key role in thrombus formation and vascular complications [9]. These comparisons underscore the clinical importance of integrating psychiatric assessments into routine thrombosis risk evaluations. Furthermore, the results from this study suggest that targeting the inflammatory and platelet activation pathways in depressed individuals could be a novel therapeutic strategy to mitigate thrombotic complications, much like the approaches used in managing other prothrombotic conditions.

The generalizability of these findings is enhanced by the inclusion of a diverse study population with well-matched depressed and control groups based on age (mean: 42.0 years), BMI (27.3±4.4 kg/m^2^), and smoking status (32.5%), ensuring that confounding factors were minimised. However, this study was conducted in a controlled clinical setting, and real-world variations in lifestyle, medication adherence, and psychiatric comorbidities could influence biomarker fluctuations and thrombosis risk. Future research should aim to replicate these findings in larger, more heterogeneous populations and explore potential therapeutic interventions, such as anti-inflammatory agents, platelet inhibitors, or structured psychiatric care programs, to mitigate the observed risks.

These findings provide a strong foundation for re-evaluating thrombosis risk assessment in psychiatric populations. Integrating psychiatric and cardiovascular screening in clinical practice may enhance early detection and prevention strategies, potentially reducing the incidence of thromboembolic complications in individuals with depression. Moving forward, interventional studies exploring pharmacological (anti-inflammatory and antiplatelet therapy) and behavioural (exercise, cognitive therapy) strategies are warranted further to delineate the impact of depression on vascular health.

## Conclusion

This study provides robust evidence that depression is strongly associated with an increased risk of thrombosis, mediated through systemic inflammation, platelet hyperreactivity, and coagulation dysregulation. Biomarker analysis revealed significantly elevated levels of IL-6 (5.8±1.2 pg/mL), TNF-α (4.2±1.1 pg/mL), and CRP (3.5±0.8 mg/L), indicating a chronic inflammatory state. Elevated D-dimer (750±150 ng/mL) and fibrinogen (450±100 mg/dL) levels further confirmed a pro-thrombotic environment in depressed individuals. The platelet activation assessment demonstrated a heightened risk for clot formation, with significantly increased expression of CD62P (65.2%), PAC-1 binding (55.4%), and GPIIb/IIIa activation (72.1%) in the depressed group. Longitudinal analysis indicated a partial resolution of inflammatory and coagulation markers over 12 months, yet biomarker levels remained significantly elevated compared to controls, emphasising the need for long-term monitoring. Multivariate regression analysis confirmed that depression severity, IL-6 levels, and platelet activation were independent predictors of thrombosis risk (p<0.001), reinforcing the necessity of integrating psychiatric evaluations into thrombosis risk assessment frameworks.

### Recommendations

Given the strong association between depression and thrombotic risk, several key recommendations emerge from this study:

- Routine Thrombosis Screening: Clinicians should consider regular cardiovascular and coagulation assessments for individuals with moderate to severe depression.
- Multidisciplinary Care Approach: Thus, integration of psychiatric and cardiovascular care might optimally facilitate early diagnosis and preventive handling of thrombosis risk.
- Targeted Therapeutic Strategies: Further study is warranted regarding the use of antiinflammatory agents, platelet inhibitors and anticoagulants in high-risk psychiatric populations.
- Lifestyle and Behavioural Interventions: As a complement to pharmacologic therapy, structured exercise programs [28] or dietary modifications or cognitive behavioural therapy (CBT) should be an option for minimisation of inflammation and vascular dysfunction in depression.

### Future work

While this study provides valuable insights, several avenues for future research remain:

- Long-Term Prospective Studies: Depression effects on thrombosis risk are dependent on prolonged observation periods beyond 12 months.
- Interventional Trials: They warrant clinical trials of anti-inflammatory and antiplatelet therapies to reduce thrombosis risk in depressed individuals.
- Mechanistic Studies: This suggests that further research is necessary to identify exactly how depression, inflammation and coagulation abnormalities are linked.
- Personalised Medicine Approaches: Genetic and epigenetic contributions that predispose psychiatric populations to thrombosis deserve to be investigated in future studies so that they can be exploited for precision medicine interventions.

### Final thoughts

This study stresses that depression is a syndrome with potentially lethal haematological and cardiovascular consequences and should no longer be restricted to a psychiatric disorder. Inflammation and coagulation biomarkers elevation and high platelet reactivity are observed, which supports that there is an effect on direct vascular health and mental health. The results of these findings suggest that psychiatric evaluations can and should be incorporated into thrombosis risk assessments for at-risk populations. Future studies should therefore seek to find effective ways of treating and preventing the adverse effects depression has on thromboembolic consequences. This study helps fill a knowledge gap in depression and its systemic effects by bringing together experts in cardiovascular disease and mental health.

## Dodatak

### Conflict of interest statement

All the authors declare that they have no conflict of interest in this work.
